# Roles of 2-oxoglutarate oxygenases and isopenicillin N synthase in β-lactam biosynthesis

**DOI:** 10.1039/c8np00002f

**Published:** 2018-05-29

**Authors:** Patrick Rabe, Jos J. A. G. Kamps, Christopher J. Schofield, Christopher T. Lohans

**Affiliations:** a Department of Chemistry , Chemistry Research Laboratory , University of Oxford , 12 Mansfield Road , Oxford , OX1 3TA , UK . Email: christopher.schofield@chem.ox.ac.uk ; Email: christopher.lohans@chem.ox.ac.uk

## Abstract

Covering: up to 2017

2-Oxoglutarate (2OG) dependent oxygenases and the homologous oxidase isopenicillin N synthase (IPNS) play crucial roles in the biosynthesis of β-lactam ring containing natural products. IPNS catalyses formation of the bicyclic penicillin nucleus from a tripeptide. 2OG oxygenases catalyse reactions that diversify the chemistry of β-lactams formed by both IPNS and non-oxidative enzymes. Reactions catalysed by the 2OG oxygenases of β-lactam biosynthesis not only involve their typical hydroxylation reactions, but also desaturation, epimerisation, rearrangement, and ring-forming reactions. Some of the enzymes involved in β-lactam biosynthesis exhibit remarkable substrate and product selectivities. We review the roles of 2OG oxygenases and IPNS in β-lactam biosynthesis, highlighting opportunities for application of knowledge of their roles, structures, and mechanisms.

## Abstract 2

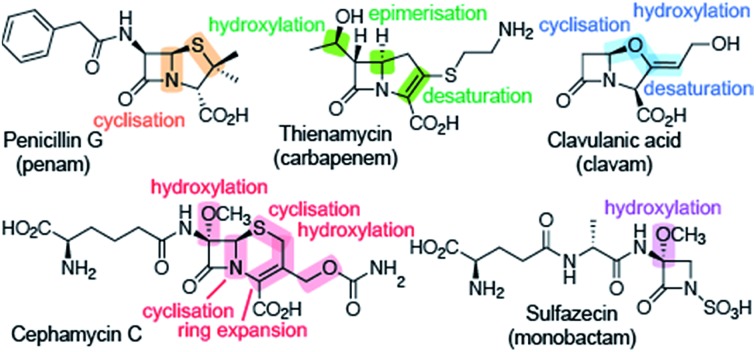
The 2OG oxygenases and IPNS contribute to the great structural diversity of β-lactam natural products, employing some remarkable mechanisms.

## 1. Introduction

**Fig. 1:**
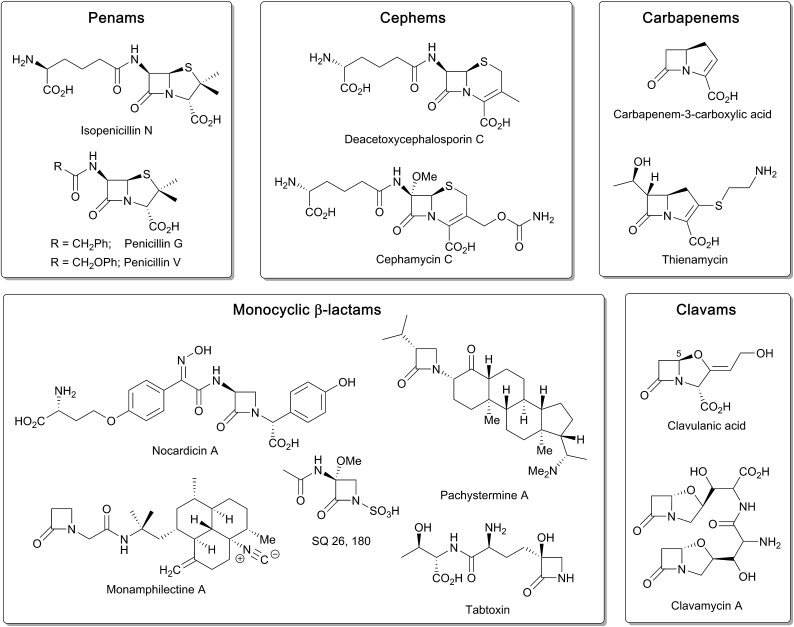
Examples of the different types of naturally occurring β-lactam ring containing molecules.

**Fig. 2:**
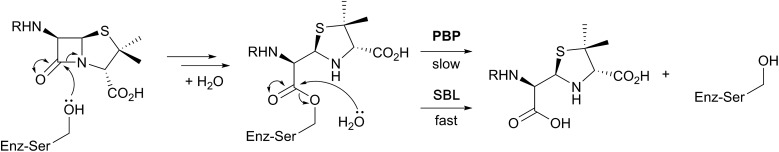
Outline interaction of β-lactams (exemplified by a penicillin) with penicillin binding proteins (PBPs) and serine β-lactamases (SBLs). A major difference between the PBPs and SBLs is the stability of the acyl-enzyme complex.

β-Lactam ring containing molecules remain amongst the most important small molecule medicines in use.^1^ Following the discovery of the penicillins in the 1940s, new generations of β-lactam antibacterials, based on natural products discovered in the 1970s and 1980s, were developed to improve their spectrum of activity and combat resistance (Fig. 1). β-Lactam antibiotics work by reacting with catalytically important nucleophilic serine residues in transpeptidases (penicillin binding proteins, PBPs) that are crucially involved in bacterial cell wall biosynthesis (Fig. 2). β-Lactam resistance is substantially, but not fully, mediated by β-lactamase enzymes which catalyse hydrolysis of the β-lactam ring (Fig. 2). The new generations of natural product-derived β-lactams include cephalosporins, carbapenems, clavams, and monocyclic β-lactams. Depending on the functionalisation patterns of the core β-lactam ring system, these compounds manifest different antibacterial activity profiles and susceptibilities to the constantly evolving β-lactamases. It should be noted that naturally occurring β-lactams have roles beyond inhibiting PBPs, sometimes with important biological consequences such as for the plant toxin tabtoxin (Fig. 1).^2^–^4^

Clavulanic acid, the sole clavam to be used clinically, has relatively weak antibacterial activity, but is a potent inhibitor of some nucleophilic serine β-lactamases. Hence, like the other clinically used β-lactamase inhibitors (sulbactam, tazobactam, and avibactam; the latter being the sole non-β-lactam approved for use as a β-lactamase inhibitor), it is used in combination with a more potent antibiotic (for example, amoxicillin and clavulanic acid are combined in the drug Augmentin).^5^ Synthetic β-lactams have found therapeutic applications other than as antibacterials.^6^ Despite extensive resistance, the longevity of β-lactams as antibacterials is remarkable in the history of anti-infective medicine.^7^ This has led to the proposal that the combination of β-lactam inhibitors of PBPs and their PBP targets is special from a chemical perspective.^7^ Thus, some naturally occurring β-lactams, which likely have ancient origins in bacteria, may well be highly evolved to their antibacterial roles. If this is the case it would seem likely that their biosynthesis may also be highly evolved – at least for secondary metabolites.

**Fig. 3:**
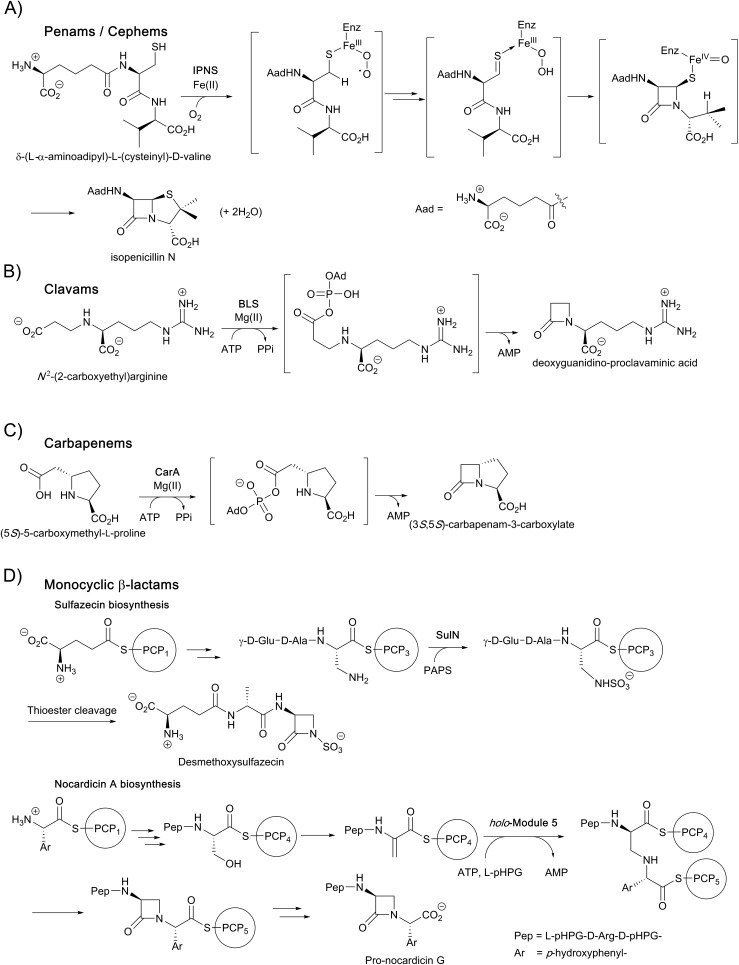
Enzymes catalysing β-lactam formation during antibiotic biosynthesis, including: (A) isopenicillin N synthase (IPNS), (B) β-lactam synthetase (BLS), (C) CarA, and (D) non-ribosomal peptide synthetases (NRPS) SulN and NocB. Outline mechanisms/steps are shown. Note that not all cofactors, co-substrates, and products are shown. PAPS: 3'-phosphoadenosine-5'-phosphosulfate; PCP: peptidyl-carrier protein.

Whether or not β-lactams are chemically unique as antibacterials, at least from an enzymology perspective, some of the reactions occurring during β-lactam biosynthesis are spectacular (for prior relevant reviews on β-lactam biosynthesis, see ref. 4 and 8–25). Although non-oxidative reactions play crucial roles in β-lactam biosynthesis, reactions catalysed by ferrous iron and 2-oxoglutarate (2OG) dependent oxygenases and the related oxidase isopenicillin N synthase (IPNS) are of particular interest (Fig. 3). The 2OG oxygenases were first discovered during work on collagen biosynthesis and their involvement in β-lactam biosynthesis was unexpected. They typically catalyse two electron oxidations, most commonly hydroxylations, in which substrate oxidation is coupled to conversion of 2OG and dioxygen to succinate and carbon dioxide.^22^

In the case of the non-penicillin β-lactam biosynthetic pathways, 2OG oxygenases play roles in diversifying the chemistry, and hence the activities, of the β-lactams formed by non-oxygen dependent chemistry. During biosynthesis of the clavam and the carbapenem bicyclic ring systems, β-lactam formation is catalysed by asparagine synthetase related enzymes (Fig. 3).^4^,^8^,^13^,^16^,^22^ These β-lactam synthetases catalyse formation of the β-lactam ring from appropriate β-amino acid precursors, *i.e.* in effect they catalyse the reverse of β-lactamase catalysis (Fig. 2), but use ATP to activate the carboxylic acid. In the case of the clavams, a monocyclic β-lactam ring is formed (Fig. 3); this acts as a precursor for the formation of the bicyclic clavam ring in reactions catalysed by the 2OG oxygenase clavaminic acid synthase (CAS). In the carbapenem pathways, β-lactam synthetases catalyse formation of the (3*S*,5*S*)-carbapenam ring system which, at least in the pathway to carbapen-2-em-3-carboxylate, is epimerised and desaturated by CarC, a 2OG oxygenase, to give a (5*R*)-carbapenem (Fig. 3). Thus, in these pathways, 2OG oxygenases play roles both in forming a bicyclic β-lactam ring system (clavams) and in modifying a bicyclic β-lactam ring system (carbapenems) to create antibacterials.^4^,^8^,^13^,^16^,^18^–^22^

Some monocyclic β-lactams, for example the nocardicins and monobactams (Fig. 3), are formed by the action of non-ribosomal peptide synthetases (NRPSs) which catalyse formation of the N1–C4 β-lactam bond from an appropriate synthetase-bound precursor peptide.^11^ In some cases, for example the monobactams and tabtoxin, 2OG oxygenases (likely) are involved in modifications subsequent to β-lactam formation.^4^

Notably, both the clavam and carbapenem biosynthetic pathways involve multiple steps en route to the clinically useful bicyclic antibiotics/β-lactamase inhibitors, for example, thienamycin and clavulanic acid (Fig. 1). Further, these pathways can result in multiple coproducts. Although it was possible to optimise clavulanic acid fermentation for commercial use,^10^ this has not yet been possible in the case of clinically useful carbapenems, which are instead produced by total synthesis.^4^ By contrast to the, at least superficially, relatively complex carbapenem and clavam biosynthesis pathways, the pathway leading to the penicillins and subsequently formed cephalosporins appears to be a paradigm of biosynthetic efficiency (Fig. 3).

Following pioneering work identifying the tripeptide δ-(l-α-aminoadipoyl)-l-(cysteinyl)-d-valine (ACV) as a precursor of the penicillins,^26^,^27^ the ferrous iron dependent oxidase IPNS was isolated and shown to catalyse the four electron oxidation of ACV to form, in a single step, the bicyclic nucleus of isopenicillin N (IPN), the first formed β-lactam in the pathway. IPN is a branchpoint in some organisms; its δ-(l-α-aminoadipoyl)-side chain can be exchanged (by the action of an amidohydrolase/acyltransferase) for a more hydrophobic side chain to give a readily extractable clinically useful penicillin, *e.g.* penicillins G or V (Fig. 1). These penicillins with hydrophobic sidechains can be used as starting points for the production of penicillins with clinically better sidechains, *via* enzymatically produced 6-aminopenicillanic acid (6-APA), or for the synthesis of cephalosporins, *via* the oxidative chemical ring expansion of the penam nucleus to that of the cephems. This chemical ring expansion of penam sulphoxides to the cephems has echoes in the biosynthetic formation of the cephalosporins (see below).

During cephalosporin biosynthesis, the side chain of IPN is epimerised to give penicillin N, which is the substrate for deacetoxycephalosporin C synthase (DAOCS), a 2OG oxygenase catalysing an unusual oxidative rearrangement to give deacetoxycephalosporin C (DAOC).^4^,^8^,^16^,^18^–^22^ Studies on the chemically interesting mechanisms and structures of IPNS and DAOCS were pioneering for the 2OG oxygenase superfamily.^28^–^31^ The procollagen prolyl hydroxylases were the first 2OG oxygenases to be identified; however, structural studies on the (animal) procollagen prolyl hydroxylases have been difficult. Unexpectedly, crystallographic studies on IPNS and 2OG oxygenases acting on small molecules^22^ led to the discovery that 2OG oxygenases are very widely distributed in aerobic biology (archaea being a likely exception), including in humans where there are approximately 60 such enzymes.^22^ Work over the last two to three decades has shown that 2OG oxygenases in humans and other animals have diverse roles. In addition to collagen biosynthesis,^32^ these enzymes are involved in epigenetics, hypoxia sensing, chromatin regulation, DNA repair, ribosome/RNA modifications, and lipid biosynthesis.^33^–^36^ By contrast with some of their reactions in β-lactam biosynthesis, to date, the reactions assigned for 2OG oxygenases in animals involve chemically simple hydroxylations and *N*-methyl demethylations (also proceeding *via* initial hydroxylation).^21^

This brief review focuses on the roles of IPNS and 2OG oxygenases in β-lactam biosynthesis, describing current mechanistic knowledge and highlighting opportunities for the generation of new antibiotics and for the improved production procedures for existing antibiotics.

## 2. Isopenicillin N synthase

The discovery of isopenicillin N synthase,^27^,^37^ and its marvellous reaction that enables oxidative ring closure to the densely functionalised heterocyclic penicillin ring system, illustrates the power of biosynthesis. IPNS catalyses formation of the fused β-lactam and thiazolidine core of the penicillins *via* the four electron oxidation of a peptide precursor with concomitant reduction of dioxygen to two water molecules.^22^,^38^,^39^ The IPNS substrate, *i.e.* the tripeptide δ-(l-α-aminoadipoyl)-l-(cysteinyl)-d-valine (ACV) was identified more than 50 years ago.^40^ ACV is biosynthesised by action of the non-ribosomal peptide synthetase ACV synthetase from the precursors l-α-aminoadipic acid, l-cysteine, and l-valine, the stereochemistry of the latter being inverted during peptide formation.^41^–^43^

**Fig. 4:**
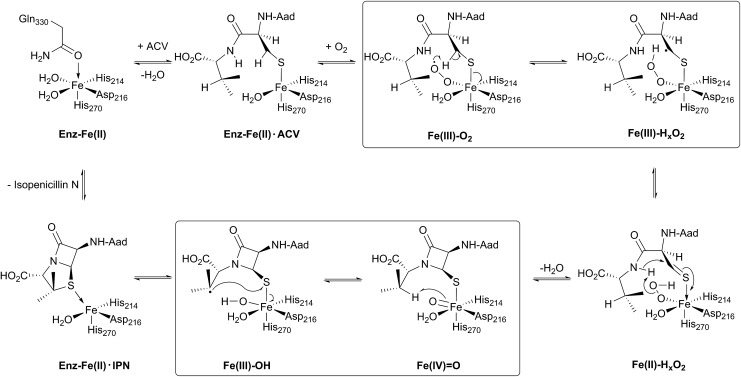
Schematic overview of the currently proposed mechanism for isopenicillin N synthase (IPNS). Mechanism adapted from ref. 38. The high-spin Fe(iii)–superoxo [Fe(iii)–O_2_^–^] and high-spin Fe(iv)–oxo [Fe(iv)=O] intermediates have been observed spectroscopically.

**Fig. 5:**
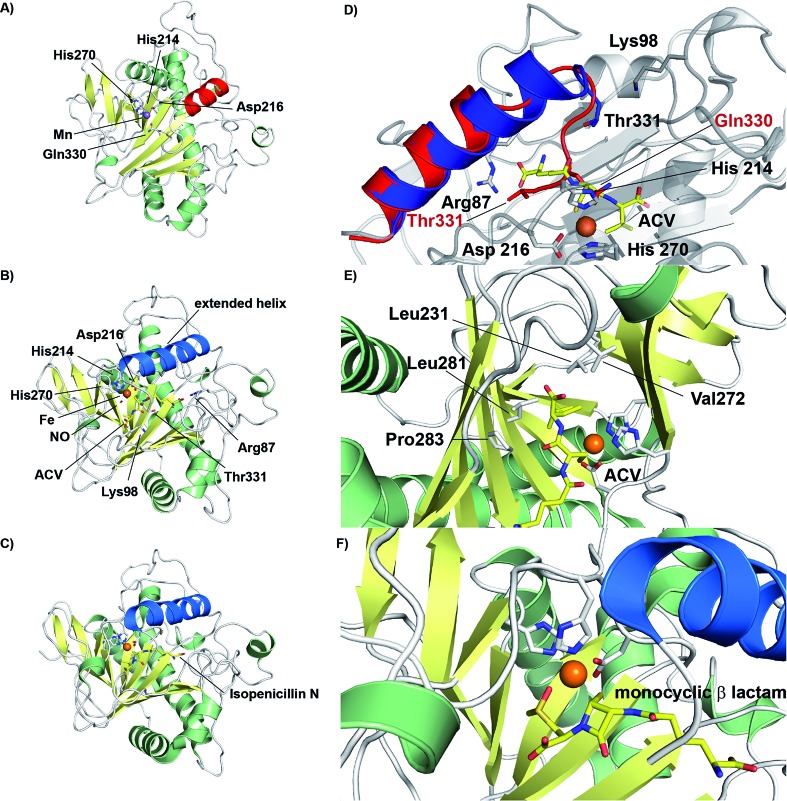
Views from crystal structures of isopenicillin N synthase. (A) An IPNS–Mn complex (PDB 1IPS)^28^ showing coordination of metal in resting state by His214, His270, Asp216, as well as by the side-chain of Gln330 (Mn, substituting for Fe, in purple); (B) the IPNS–Fe–ACV–NO complex (PDB ; 1BLZ),^29^ showing binding of ACV by Arg87, the thiolate–Fe interaction, and enclosure of ACV at the active site, including by the extended helix; Thr331 and Lys98 form a salt bridge to constrain the conformation of the complex and to shield the active site/substrate (Fe in orange, extended helix in blue); (C) the IPNS–Fe–IPN complex (PDB ; 1QJE);^55^ (D) expanded view of the superposition of the IPNS–Fe–ACV complex active site [PDB ; 1BLZ; showing enclosure of ACV by the extended helix (in blue)] with the substrate-free IPNS–Mn complex (PDB ; 1IPS; α-helix indicated in red);^29^ (E) hydrophobic residues bind the ACV valine isopropyl group;^29^,^61^ (F) the IPNS–Fe–monocyclic β-lactam complex (PDB ; 1QJF)^55^ formed after incubation with δ-(l-α-aminoadipoyl)-l-(cysteinyl)-*S*-methyl-d-cysteine (ACMC) and O_2_, supporting initial β-lactam ring formation during stepwise bicyclisation to isopenicillin N.

The current detailed understanding of the mechanism of IPNS is based on extensive spectroscopic,^44^–^46^ computational,^47^–^50^ crystallographic,^28^,^29^,^51^–^58^ and substrate analogue studies (Fig. 4). Here we summarise these results – see earlier reviews for detailed descriptions of IPNS.^4^,^6^,^15^,^17^,^21^–^24^,^39^ The first crystal structure of IPNS (from *Aspergillus nidulans*) was obtained in complex with manganese substituting for iron; this structure revealed that in the resting enzyme state, the single active site metal is coordinated by two histidine (His214 and His270) and one aspartate residue (Asp216) as well as by the side-chain of Gln330 (Fig. 5A).^28^ The overall fold of IPNS is based on a distorted double-stranded β-helix (DSBH or ‘jelly roll’) core; this scaffold supports the triad of Fe(ii) binding residues, and was subsequently shown to be conserved in 2OG oxygenases, including DAOCS.^53^,^59^ As shown in subsequent anaerobic structures obtained in the presence of ACV and Fe(ii), Gln330 (ref. 28 and 60) (and one iron coordinating water) is displaced from the active site on binding of ACV, triggering conformational rearrangement of the *C*-terminal region (Asn326-Thr331).^29^ Together with other conformational changes, this arrangement means that the iron-ligated ACV is enclosed at the active site (Fig. 5B).^29^,^57^,^61^ Conformational changes at the active site upon binding of ACV are shown in Fig. 5D. Recently reported results for a *C*-terminally truncated IPNS variant support the proposal that the *C*-terminal α-helix shields the active site and hinders (potential) side reactions during catalysis.^61^

In the IPNS·ACV·Fe(ii) complex, ACV coordinates to the Fe(ii) *via* its cysteinyl thiolate and appears conformationally constrained in a manner appropriate for β-lactam ring formation. In addition to metal complexation, ACV is bound by both hydrophobic and electrostatic interactions, the latter including the interaction of the l-α-aminoadipoyl carboxylate group with Arg-87.^29^,^51^,^61^ The valine isopropyl group is bound in a hydrophobic pocket formed by the side-chains of Pro283, Leu223, Leu231, Val272, and Thr221 (Fig. 5E). Recent mutational studies on the role of these residues highlights their importance in substrate recognition and catalysis.^61^

**Fig. 6:**
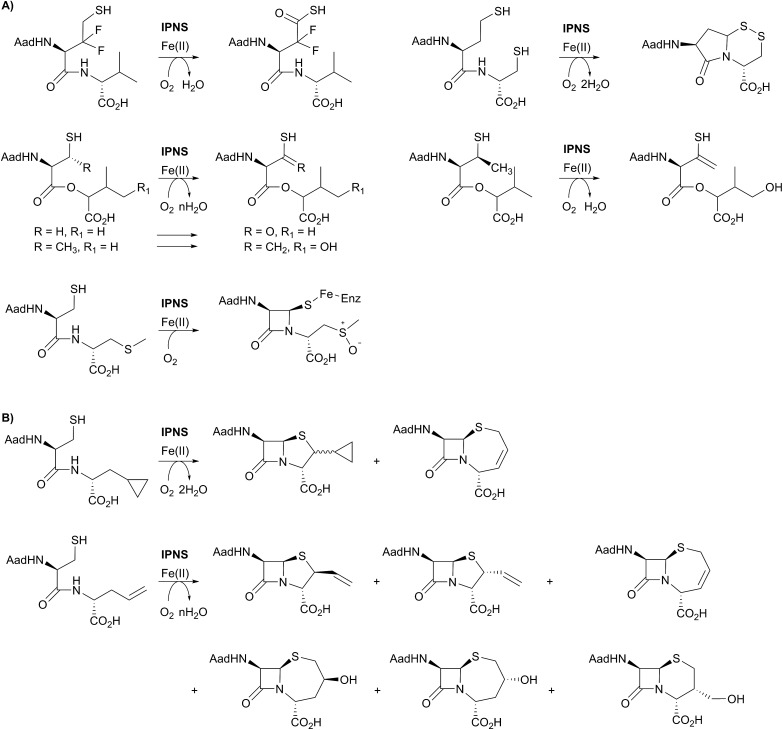
(A) Substrate analogues exemplifying the catalytic promiscuity of IPNS with respect to the type of oxidation reaction catalysed. The products derived from the depsipeptide analogues and the *S*-methyl cysteine analogue were observed crystallographically. (B) Examples showing the ability of IPNS to make different β-lactam ring systems by substituting the *C*-terminal valine of the natural (δ-(l-α-aminoadipoyl)-l-(cysteinyl)-d-valine) substrate.

Crystallographic analysis of the IPNS·ACV·Fe(ii)·NO complex^29^ showed that NO (acting as a dioxygen analogue) binds adjacent to the cysteinyl thiol with the oxygen atom of the complexed NO directed toward the cysteinyl β-carbon. These observations support formation of the β-lactam ring prior to the thiazolidine ring (Fig. 5B) and are consistent with prior kinetic studies, in particular those employing deuterium labelled ACV substrates (with [3,3-^2^H_2_]-Cys or [3-^2^H_1_]-Val labelled ACV residues).^22^,^57^ Further support for initial β-lactam ring formation came from crystallographic analyses of IPNS with the substrate analogue δ-(l-α-aminoadipoyl)-l-(cysteinyl)-*S*-methyl-d-cysteine (ACMC), which was crystallised under anaerobic conditions, then exposed to a high pressure of oxygen. The resultant structure showed formation of a monocyclic β-lactam (with oxidation of the *S*-methyl group),^55^,^56^ providing strong experimental support for initial β-lactam formation (Fig. 5F and 6A).

Mechanistic proposals for IPNS arising from early kinetics, substrate analogue, and crystallographic studies are supported by recent stopped flow, modelling, and spectroscopic (including by Mossbauer) studies.^38^ Following ACV binding/ligation to the active site Fe(ii) (involving displacement of one metal ligated water and incompletely defined induced fit processes), dioxygen binds and reacts to form an (unusually stable) iron(iii) superoxo species.^38^ The first step in the ACV-catalysed reaction involves abstraction of the pro-(*S*) cysteinyl C-3 hydrogen to give a thioalkyl radical. After inner-sphere electron transfer, a ferrous hydroperoxo intermediate [Fe(ii)–OOH] and a thioaldehyde are formed. Heterolysis of the peroxo bond to form a water molecule, concomitant with abstraction of the cysteinyl-valine amido hydrogen by the terminal peroxide oxygen, and nucleophilic attack of the amide nitrogen onto the thioaldehyde forms a monocyclic β-lactam intermediate linked to a ferryl species [Fe(iv)=O] (Fig. 4).^55^,^57^ This second key intermediate, which has been characterised by spectroscopy,^38^ mediates radical type abstraction of the ACV valine C-3 hydrogen resulting in a ferric hydroxyl [Fe(iii)–OH] species. Reaction of the valinyl radical on the sulphur enables isopenicillin N product formation and restoration of the metal to the Fe(ii) state (Fig. 4 and 5C).^55^

A feature of IPNS is its ability to accept substrate analogues, with a few selected examples shown in Fig. 6.^4^,^15^,^22^,^62^–^78^ The substrate analogue work has provided further insight into the mechanism of IPNS, and highlights the potential of non-heme oxygenases for production of synthetically challenging molecules. In terms of biocatalysis, the observation that ACV analogues lacking the amino group in the δ-(l-α-aminoadipoyl)-side chain are IPNS substrates has potential utility in pathway engineering.^4^,^15^ Some ACV analogues with hydrophobic groups substituting for the δ-(l-α-aminoadipoyl) residue of ACV are IPNS substrates, raising the possibility of engineering both ACV synthetase and IPNS to produce clinically useful penicillins in two enzyme catalysed steps.^4^,^15^

One mechanistic insight arising from the substrate analogue work is the delicate nature of the balance in regiochemistry of IPNS catalysed peptide oxidations. By way of example, δ-(l-α-aminoadipoyl)-l-(3,3-difluorohomocysteinyl)-d-valine is oxidised by IPNS giving neither bicyclic nor monocyclic products; instead the 3,3-difluorohomocysteinyl residue undergoes four electron oxidation resulting in a thiocarboxylic acid (Fig. 6A).^62^ Use of δ-(l-α-aminoadipoyl)-l-(homocysteinyl)-d-cysteine enabled trapping of a bicyclic γ-lactam as a disulfide (Fig. 6A).^63^,^78^ More recent work in the crystalline state has employed substrate analogues in which the cysteinyl-valine amide of ACV is replaced with an ester (*i.e.*, depsipeptide analogues), with particularly interesting results being obtained with β-methyl cysteinyl analogues; the depsipeptide with a cysteinyl (*3S*)-methyl group yielded a thioenol/thioketone, whereas one depsipeptide without the methyl group yielded a thiocarboxylic acid.^68^,^69^

ACV analogues employing valine substitutes strikingly reveal the potential for promiscuity in the reactions of the monocyclic β-lactam ferryl intermediate, at least with unnatural substrates (Fig. 6).^4^,^15^ Multiple products have been isolated with some unsaturated or cyclopropane ring containing valine analogues, with the balance between products being manipulated by use of deuterium labels.^15^,^22^,^63^–^66^ This promiscuity, including the ability to form ‘expanded’ ring products, is clearly manifested with replacement of the d-valine of ACV by d-allylglycine or by d-cyclopropylalanine (Fig. 6B). At least some of the observed products are formed *via* radical intermediates.^63^–^66^

## 3. 2OG oxygenases in cephalosporin biosynthesis – deacetoxycephalosporin C and deacetylcephalosporin C synthase

The committed step in cephalosporin biosynthesis is catalysed by deacetoxycephalosporin C synthase (DAOCS).^4^ Eukaryotic microorganisms have a single bifunctional 2OG oxygenase DAOCS/DACS,^79^ which has both deacetoxycephalosporin C synthase (DAOCS) and deacetylcephalosporin C synthase (DACS) activities. By contrast, in prokaryotes two distinct enzymes are highly, but incompletely, selective for the penam expansion (DAOCS)^80^ and the DAOC hydroxylation steps (DACS).^81^–^83^ Isopenicillin N is not a substrate for DAOCS, though note that δ-(d-α-aminoadipoyl)-l-(cysteinyl)-d-valine is an IPNS substrate.^4^,^84^,^85^ In microorganisms producing cephalosporins, the δ-(l-α-aminoadipoyl) side chain of isopenicillin N undergoes epimerisation to give the δ-(d-α-aminoadipoyl) side chain of penicillin N, which is the DAOCS substrate. In prokaryotes, this reaction is catalysed by a pyridoxal dependent epimerase.^86^–^90^ In eukaryotes, a different epimerisation system is present, encoded for by genes cefD1 and cefD2; the encoded protein CefD1 is proposed to catalyse CoA ester formation, while CefD2 is proposed to catalyse epimerisation.^91^,^92^

At least one 2OG oxygenase is also involved in the 7α-functionalisation of cephalosporins. Two types of 7α-functionalised cephalosporins with potent antibiotic activity are known: the cephamycins with a 7α-methoxy group, and the cephabacins with a 7α-formylamino group. Whilst the biosynthesis of the latter has not been defined, the methyl group and the oxygen of the 7α-methoxy group are derived from methionine and dioxygen, respectively.^93^ Studies with cell-free extracts of *Streptomyces clavuligerus*, using *O*-carbamoyl DAC as a substrate, showed the process is dependent on Fe(ii), 2OG, and *S*-adenosylmethionine. Evidence for sequential hydroxylation then methylation came from the isolation of the 7α-hydroxy-*O*-carbamoyl DAC. Sequencing of the cephamycin biosynthesis gene cluster suggests cmcI and cmcJ encode for a methyltransferase and a 7α-hydroxylase, respectively.^94^ The assignment of CmcI as a methyltransferase was supported by structural studies;^95^ as yet no biochemical studies on recombinant CmcJ have been reported. The improvement of 7α-methoxycephalosporins production by overexpression of cmcJ and cmcI in *S. clavuligerus* has been recently reported.^96^

**Fig. 7:**
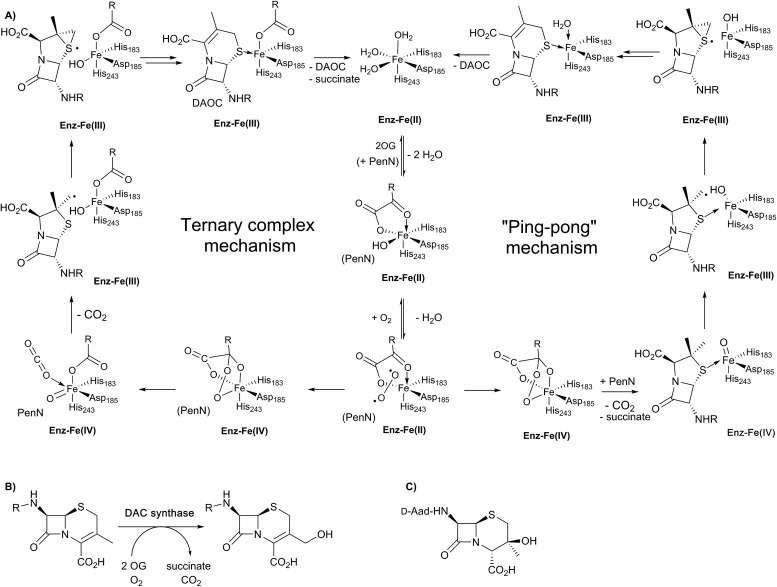
Schematic overview of proposed mechanisms for deacetoxycephalosporin C synthase (DAOCS). (A) The ternary complex mechanism shown employs the consensus process for formation of the ferryl intermediate in the presence of the substrate (penicillin N) as supported by stopped-flow kinetic assays and binding studies by non-denaturing mass spectrometry and NMR.^106^ An alternative “ping-pong” mechanism has been proposed based on crystallographic information – see text. Note that penicillin N (PenN) binds to the enzyme-Fe(ii)-2OG complex in the case of the ternary complex mechanism. (B) The allylic oxidation of deacetoxycephalosporin C (DAOC) to give deacetylcephalosporin C (DAC) as catalysed by deacetylcephalosporin C synthase (DACS). (C) Structure of the byproduct observed in DAOCS catalysis.

Insight into the DAOCS/DACS mechanisms has come from isotopic labelling experiments, spectroscopy, and crystallography.^4^,^8^,^16^,^18^,^22^,^24^,^31^ The DACS catalysed hydroxylation reaction is typical of 2OG-dependent oxygenase type reactions, albeit occurring at an allylic position (Fig. 7B); it likely proceeds *via* hydrogen abstraction involving a ferryl intermediate, generated by reaction of 2OG and dioxygen with consequent production of carbon dioxide and succinate, as proposed for other 2OG oxygenases.^4^,^8^,^22^ Consistent with this, the reaction proceeds with, at least, partial retention of stereochemistry.^97^ By contrast, the DAOCS catalysed oxidative ring expansion reaction (Fig. 7A) is unique to date in enzymology. It does, however, have precedent in the non-enzymatic oxidative rearrangement of penicillins to cephalosporins, in which a penicillin β-sulfoxide is converted into a cephalosporin where the (pro-*S*) β-methyl group of a penicillin is incorporated into the dihydrothiazine ring.^98^ Studies in cells and with isolated enzyme have shown that the β-methyl group of penicillin N forms the endocyclic C-3 carbon of DAOC (Fig. 7).^98^–^100^ By contrast with the results for the DACS reaction, cellular studies employing (3-pro-*R*)-valine, with a ^1^H/^2^H/^3^H labelled chiral methyl group at the position that forms the endocyclic C-3 of cephalosporin C, imply loss of stereochemistry during the DAOCS reaction (Fig. 7A), suggesting the involvement of a radical intermediate, as supported by biomimetic studies.^101^–^103^

Work with deuterium labelled penicillin N has shown that during the DAOCS reaction, a hydrogen is removed from the (pro-*S*) β-methyl group prior to loss of the C-3 hydrogen.^104^,^105^ Further, incubation with (3-^2^H)penicillin N resulted in a substantial increase in the observed amount of 3β-hydroxy-3α-methylcepham (Fig. 7C), which is only otherwise observed as a minor by-product.^104^,^105^ Coupled with studies on other 2OG oxygenases, these and other kinetic observations led to a proposal for DAOCS catalysis in which 2OG binding is followed by that of penicillin N. Recent spectroscopic studies support this mechanism and the formation of a ternary DAOCS·2OG·penicillin N complex.^106^ Following binding of dioxygen, oxidative decarboxylation of 2OG can occur to form carbon dioxide, succinate, and a ferryl intermediate as proposed in DACS catalysis. The ferryl intermediate can abstract a hydrogen to give a β-methyl radical which ring expands to give a cepham radical, which then loses a hydrogen atom to give DAOC (*i.e.*, the ternary complex mechanism in Fig. 7A). Deuteration at the penicillin N C-3 position favours hydroxylation to give 3β-hydroxy-3α-methylcepham (Fig. 7C), due to operation of a primary isotope effect.^104^

**Fig. 8:**
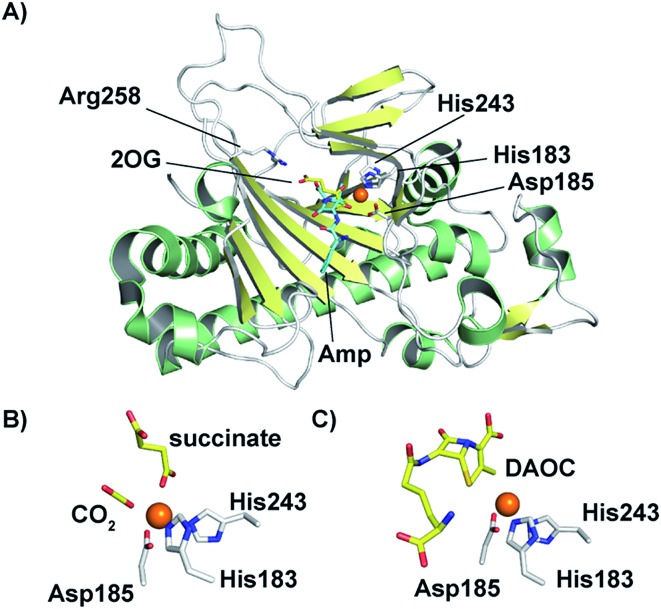
Structural views for deacetoxycephalosporin C synthase (DAOCS). (A) The overall fold (PDB 1UNB)^114^ and selected active site elements of DAOCS (including the Fe binding residues) are conserved with IPNS. (B) Active site view from a DAOCS–Fe–succinate–CO_2_ complex structure (DAOCS R307A mutant, PDB ; 1E5H);^114^ note the similar arrangement of Fe-binding ligands to that in IPNS (Fig. 5); (C) active site view from a DAOCS–Fe–DAOC product structure (PDB ; 1W2O);^114^ note the apparent overlap of the DAOC and 2OG/succinate binding sites – with other structures this led to the proposal of an unusual mechanism for DAOCS – see text and Fig. 7.

In 1998, an X-ray crystal structure of DAOCS from *S. clavuligerus*, the first of a 2OG oxygenase, was reported.^30^ The structure revealed the distorted double-stranded β-helix also present in IPNS^28^,^29^ and other 2OG oxygenases.^4^,^8^,^22^,^107^ As for IPNS,^28^,^29^ DAOCS (and by implication the other 2OG oxygenases involved in cephalosporin biosynthesis) has the conserved Fe(ii) binding triad 2-His-1-Asp motif (His183, Asp185, and His243 in DAOCS). Structures were obtained for DAOCS·Fe(ii)·2OG and DAOCS·Fe(ii)·succinate complexes. These are consistent with operation of the consensus ordered sequential mechanism for 2OG oxygenases,^107^–^111^ in which 2OG binds prior to penicillin N, and the DAOC product leaves prior to succinate (Fig. 8A and B). 2OG was observed to bind in a bidentate manner to the active site metal *via* its oxalate group with displacement of two water molecules.^30^ A second critical interaction for 2OG binding is *via* its 5-carboxylate with Arg258 which is part of a conserved RXS 2OG binding motif present in DAOCS subfamily 2OG oxygenases (including biomedicinally important human enzymes)^22^ (Fig. 8A). Mutagenesis of Arg258 (to Gln258) severely reduces DAOCS activity.^112^

Although it is predominantly monomeric in solution, DAOCS was initially crystallised as a trimer in which the *C* terminus of one protein monomer inserts into the active site of its neighbour monomer;^30^ this crystal form is likely not amenable to obtaining substrate complexes. Mutagenesis/truncation of the *C*-terminus yielded a catalytically active form of DAOCS which crystallises as a monomer.^113^,^114^ Structures were obtained of complexes with the (poor) substrate analogue penicillin G and ampicillin as well as one with the product DAOC (Fig. 8C).^114^ Structures of DAOCS-Fe(ii)·penicillin G·2OG and DAOCS·Fe(ii)·ampicillin·2OG revealed apparent overlapping of the penicillin substrate analogue with the co-substrate (2OG) binding sites. These results were interpreted as supporting an unprecedented “ping-pong” mechanism in which 2OG and dioxygen react to give a ferryl intermediate, succinate leaves, and only then does penicillin N bind with subsequent ring expansion as proposed above (Fig. 7A).^114^ However, more recent studies on DAOCS pre-steady-state kinetics and binding studies with different substrate analogues^106^ do not support such a mechanism, instead suggesting that the formation of a ternary DAOCS·Fe(ii)·2OG·penicillin substrate complex occurs after 2OG binding. Future studies on DAOCS could focus on characterising details of the proposed radical mechanism of ring expansion.

The polar δ-(d-α-aminoadipoyl)-side chain of naturally occurring cephalosporins is not clinically useful and makes them difficult to extract. In order to enable production of cephalosporins with hydrophobic (or, potentially clinically useful side-chains) which are easier to purify, approaches guided by structural studies or directed evolution to engineer the natural cephalosporin biosynthesis pathway are being employed.^112^,^115^–^131^ One approach involves using mutagenesis to alter the selectivity of DAOCS such that it efficiently accepts penicillins with hydrophobic side-chain (*e.g.* penicillin G) rather than the natural δ-(d-α-aminoadipoyl) side chain. The main focus of recent studies has been on modifications in the *C*-terminal region of DAOCS, which is involved in substrate recognition.^118^,^123^,^131^

## 4. The role of clavaminic acid synthase (CAS) in clavam biosynthesis

The biosynthetic pathways leading to clavams contrast with those for the penicillins and cephalosporins, both with respect to β-lactam ring formation and functionalisation processes. The initial steps of clavam biosynthesis are believed to occur *via* a conserved pathway leading to (3*S*,5*S*)-clavaminic acid, which acts as a branchpoint for diverging pathways yielding (3*R*,5*R*)-clavulanic acid and the (5*S*)-clavams.^132^ The formation of clavaminic acid involves the remarkable 2OG oxygenase clavaminic acid synthase (CAS), which catalyses three distinct oxidations, *i.e.*, hydroxylation, cyclisation, and desaturation reactions.

The committed step in clavam biosynthesis comprises reaction of l-arginine with glyceraldehyde-3-phosphate, yielding the β-amino acid *N*^2^-(2-carboxyethyl)arginine (CEA), as catalysed by the thiamine pyrophosphate enzyme, carboxyethylarginine synthase (CEAS). The (3*S*,5*S*) β-lactam core of the clavams is next formed *via* an ATP-dependent reaction catalysed by a β-lactam synthetase (BLS). CAS then catalyses the hydroxylation of deoxyguanidino-proclavaminic acid, yielding guanidino-proclavaminic acid, in a reaction typical of 2OG oxygenases.^133^ Prior to further oxidation by CAS, proclavaminate amidino hydrolase (PAH) catalyses hydrolysis of the guanidino group of guanidino-proclavaminic acid, forming proclavaminic acid.^134^–^136^ CAS then catalyses two further reactions: first the unusual bicyclisation of proclavaminic acid to form dihydroclavaminic acid, then a desaturation reaction to form clavaminic acid.^137^ The subsequent steps leading to clavulanic acid, in which the amino group of (3*S*,5*S*)-clavaminic acid is replaced with a hydroxyl group and the two stereocenters are epimerised, have not been fully elucidated.^138^ The final step in the pathway involves NADPH/NADH dependent reduction of (3*R*,5*R*)-clavaldehyde to give clavulanic acid.^139^–^141^ Another oxygenase, potentially a P450 type enzyme, may be involved in the installation of the allylic hydroxyl oxygen of clavulanic acid, as growth of the producing organism in an ^18^O_2_ atmosphere resulted in isotopic labeling at this position.^142^

**Fig. 9:**
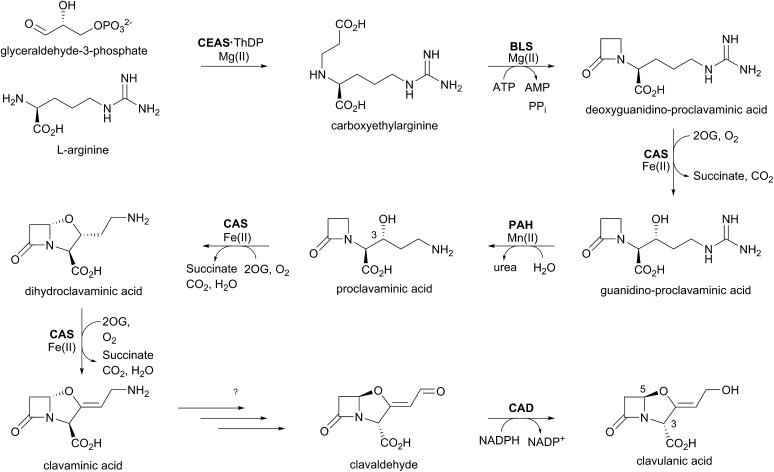
Biosynthetic scheme for clavulanic acid formation showing the trifunctional role of clavaminic acid synthase (CAS). Note that not all co-factors, co-substrates, and products are shown. CEAS: carboxyethylarginine synthase; BLS: β-lactam synthetase; PAH: proclavaminate amidino hydrolase; CAD: clavaldehyde dehydrogenase; ThDP: thiamine diphosphate.

Whilst investigating the cell-free production of clavulanic acid, Elson *et al.* discovered the production of clavaminic acid and proclavaminic acid by a clavulanate-producing strain of *S. clavuligerus*.^134^ They also described the isolation of native CAS from this strain, which catalysed the conversion of proclavaminic acid into clavaminic acid (Fig. 9). CAS was shown to be a 2OG oxygenase, with conversion of proclavaminic acid into clavaminic acid requiring two equivalents of 2OG. Incubation of ^13^C-labeled proclavaminic acid or clavaminic acid with the clavulanic acid producer resulted in isotopic incorporation into clavulanic acid, indicating their roles as intermediates in clavulanic acid biosynthesis.^143^

**Fig. 10:**
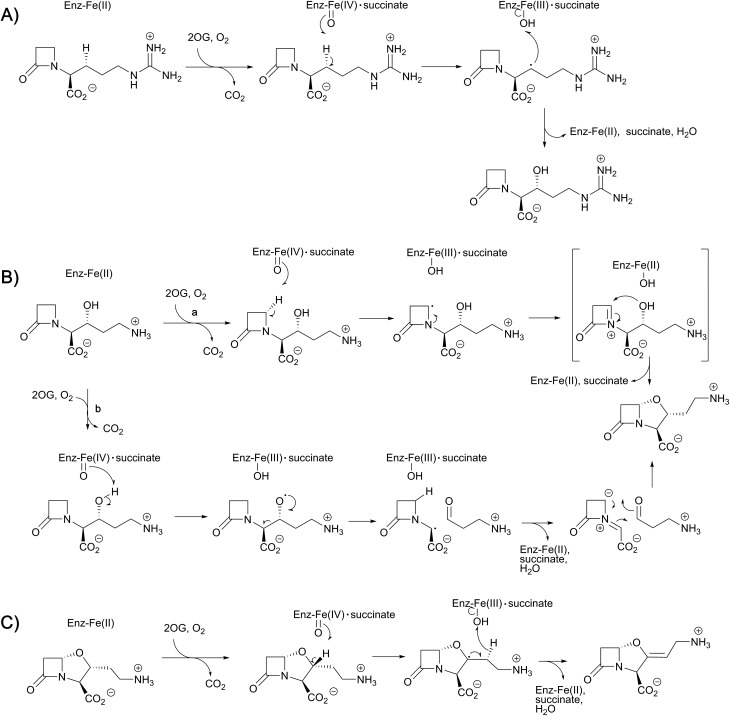
Proposed outline mechanisms for the three different reactions of clavaminic acid synthase (CAS). (A) Stereoselective hydroxylation of deoxyguanidino-proclavaminic acid – a typical reaction for the 2OG oxygenases; (B) two possible mechanisms (a and b) for the oxidative cyclisation of proclavaminic acid; and (C) the desaturation of dihydroclavaminic acid.

While monitoring the conversion of proclavaminic acid into clavaminic acid by CAS, the presence of a minor species was observed by ^1^H NMR;^135^ incubation of CAS with C-3 deuterium-labeled proclavaminic acid resulted in accumulation of this species, apparently due to a primary isotope effect, allowing for its isolation and characterisation as dihydroclavaminic acid (Fig. 9). This indicated that the cyclisation reaction catalysed by CAS, wherein the oxazolidine ring is formed, precedes the CAS-catalysed desaturation reaction. It was subsequently demonstrated that CAS catalyses the efficient hydroxylation of deoxyguanidino-proclavaminic acid to give guanidino-proclavaminic acid,^133^ which is the substrate for PAH. Studies employing an affinity label targeting CAS implied that all three reactions were catalysed by a single active site and required only a single iron center.^141^,^142^ Although the ability of a single active site to catalyse multiple reactions is precedented with other 2OG oxygenases,^22^ CAS is remarkable because of the three distinct types of reactions it catalyses (Fig. 10). Further, one of these – the first hydroxylation – occurs with a guanidine side chain, whereas the latter two – bicyclisation and desaturation – occur with an amino side chain. Thus, the role of PAH appears to be to substitute the substrate side chain in order to enable CAS to catalyse two additional reactions. Note the amino analogue of proclavaminic acid is neither an efficient inhibitor, nor a substrate of CAS.^134^

**Fig. 11:**
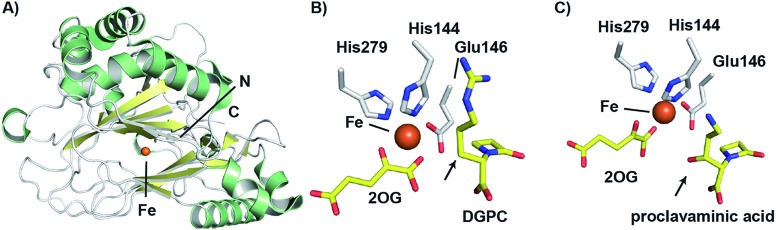
Views from structures of clavaminic acid synthase (CAS). (A) View from a crystal structure of CAS1 (PDB 1DS1)^145^ highlighting the core double-stranded β-helix fold (yellow). (B) View of the CAS1 active site with bound deoxyguanidinoproclavaminic acid (DGPC; PDB ; 1GVG),^147^ showing the orientation of the position to be hydroxylated (C-3) towards the metal (arrowed). Use of a Glu (rather than the more typical Asp) to bind to the iron may contribute to the catalytic flexibility of CAS. Note this structure was obtained using NO in place of O_2_. (C) View from the active site of a crystal structure of CAS1 with proclavaminic acid (PDB ; 1DRT).^145^ Note that the hydroxyl group is oriented towards the iron (arrowed), consistent with a mechanism in which the hydroxyl group hydrogen atom is abstracted by a ferryl species.

The combined crystallographic and solution studies have yielded insight into the mechanisms of CAS. A crystal structure of the complex of CAS1 (one of two CAS isozymes in *Streptomyces* spp.^136^) with Fe(ii) and 2-OG reveals the characteristic 2OG oxygenase core fold based on a distorted double stranded β-helix fold, consisting of eight β-strands, enveloped by two α-helical regions (Fig. 11).^144^ While there is little overall sequence similarity between CAS and the penicillin/cephalosporin β-lactam biosynthesis enzymes, DAOCS/DACS and IPNS, the similar overall structure and arrangement of active site motifs implies that, as with all characterised 2OG oxygenases, they ultimately arise from divergent evolution. There is more sequence similarity between CAS and the 2OG oxygenases of carbapenem biosynthesis, including CarC,^145^ suggesting a closer evolutionary relationship between these two sets of 2OG oxygenases.

The CAS structures revealed that iron binding is mediated by the side chains of His144, Glu146, and His279; the presence of an HXE···H motif in CAS rather than an HXD···H motif, as in IPNS and DAOCS is notable. The presence of a glutamyl residue in this motif, in place of the more commonly observed aspartyl residue is proposed to be related to the ability of CAS to catalyse multiple reactions in a single active site.^145^ As glutamyl residues are longer than aspartyl residues by a methylene unit, the position of the iron center may be relatively more flexible, which may permit the enzyme to catalyse different oxidation reactions with its three structurally distinct substrates.

As with other 2OG oxygenases (*e.g.* DAOCS – see above),^106^ bidentate binding of 2OG to the metal center is proposed to be followed by that of substrate proximate to the metal, an event which weakens binding of a metal-ligated water, so promoting ligation of dioxygen to the metal. An ordered sequential mechanism is supported by spectroscopic studies with CAS.^146^ Notably, the orientations of water and the 2OG 1-carboxylate around the metal center were noted to be reversed relative to what was observed in the DAOCS crystal structure. Studies using nitric oxide, as a mimic of dioxygen, revealed loss of a metal ligated water and rearrangement of the 2OG C-1 carboxylate on nitric oxide complexation.^147^ The precise mechanistic significance of this for CAS catalysis has yet to be defined, but analogous rearrangements are proposed with some other 2OG oxygenases, but in other cases (*e.g.* DAOCS) appear not to operate.^24^

Crystal structures have also been solved for CAS complexed with its substrates proclavaminic acid and deoxyguanidinoproclavaminate (DGPC), as well as the DGPC mimic *N*-acetylarginine.^145^,^147^ The binding positions observed for DGPC and *N*-acetylarginine are such that the 3-pro-*R* hydrogen atom is oriented towards the metal center, apparently allowing for its abstraction by the ferryl intermediate (Fig. 11B).^145^,^147^ The guanidino group of DGPC (and *N*-acetylarginine) appears to be snugly bound at the active site, being oriented to interact with the side chains of two aspartyl residues, while the β-lactam ring (or *N*-acetyl group) is positioned in a hydrophobic pocket.^145^,^147^ Thus, the CAS catalysed hydroxylation reaction appears to be enabled by precise enzyme-substrate interactions, consistent with solution studies on the high degree of stereoselectivity for the CAS catalysed hydroxylation of DGPC and NAA.^148^

While Arg297 is positioned so as to interact with the carboxylate of DGPC, the position of this residue is shifted somewhat in the structure with proclavaminic acid (Fig. 11C) so as to hydrogen bond with the hydroxyl group of the substrate for bicyclisation.^145^,^147^ Further, the amino group of proclavaminic acid does not appear positioned to interact directly with the aspartyl residues which are involved in binding to the guanidino group of the DGPC, but may interact with them through water molecules.^145^,^147^ Overall, the available structures imply proclavaminic acid is bound less precisely in the active site – possibly reflecting conformational changes during dihydroclavaminic acid formation. Studies using labelled proclavaminic acid have shown selective loss of the 4-pro-*R* β-lactam hydrogen during oxazolidine ring formation, *i.e.* reaction occurs with retention of stereochemistry.^149^ Modelling studies have led to the proposal that the ferryl intermediate first abstracts a hydrogen from the alcohol of proclavaminic acid; 1,5-hydrogen abstraction may then remove the β-lactam C-4 hydrogen so enabling subsequent oxazolidine ring formation, potentially *via* a radical retro aldol fragmentation followed by a 1,3-dipolar reaction (Fig. 10).^150^ Information on the CAS-catalysed desaturation is hindered by lack of a dihydroclavaminic acid complex structure, but solution studies reveal the CAS reaction proceeds with retention of stereochemistry^141^ and a stepwise process seem most probable (Fig. 10).

Given the clinical importance of clavulanic acid as an inhibitor of serine β-lactamases, there is interest in exploiting the clavam biosynthetic pathway to generate clavulanic acid analogues. Studies with CAS have demonstrated flexibility in terms of the substrates accepted by this enzyme, perhaps unsurprisingly given the catalytic flexibility of this enzyme. Notably, given the development of avibactam,^7^,^151^–^154^ CAS is able to generate a γ-lactam derivative of clavaminic acid from the γ-lactam variant of proclavaminic acid.^155^ Furthermore, CAS stereospecifically hydroxylates DGPC substrate analogues lacking a β-lactam ring, *e.g. N*-acetylarginine and *N*-acetylornithine, with alkene formation being observed in some cases, reflecting the flexibility in CAS catalysis.^133^,^141^,^148^ However, as the biosynthetic steps leading from clavaminic acid to clavulanic acid are unclear, it is also unclear whether the enzymes involved in these steps would tolerate such substitutions at an early stage in the pathway. Hence, engineering the end of the biosynthesis pathway to add functional groups to clavulanic acid may be more productive.

## 5. 2OG oxygenases in carbapenem biosynthesis

Biosynthetic studies of the carbapenems have primarily focused on the simplest carbapenem, (5*R*)-carbapen-2-em-3-carboxylic acid, and the more structurally complex and potent antibiotic thienamycin.^4^,^11^,^13^ The biosynthetic pathways to these two carbapenems may initially follow a common pathway, but diverge in later steps involving the desaturation and epimerisation of the five-membered ring, as well as the functionalisation at C-2 and C-6 as present in thienamycin.^4^,^11^,^13^,^156^,^157^

**Fig. 12:**
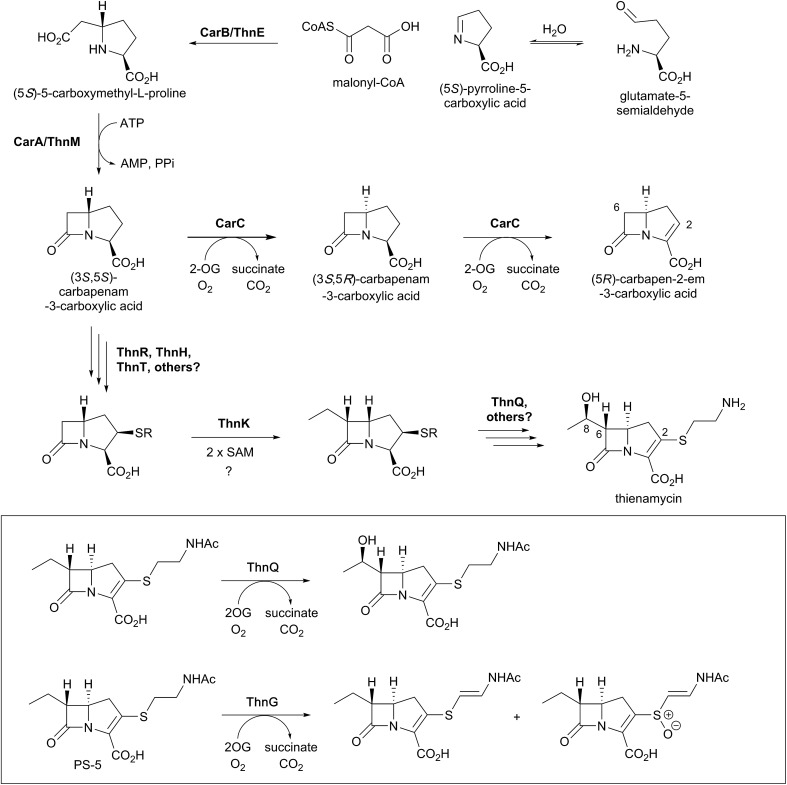
Biosynthetic scheme for carbapenem formation showing the roles of 2OG oxygenases. Carbapenem synthase (CarC) catalyses both epimerisation and desaturation steps in the biosynthesis of (5*R*)-carbapen-2-em-3-carboxylic acid. ThnQ and ThnG have (at least) roles in carbapenem side chain modification during thienamycin biosynthesis. Note that not all co-factors, co-substrates, and products are shown.

The committed step of both pathways likely involves a decarboxylative aldol type reaction of malonyl-CoA with pyrroline-5-carboxylate, as catalysed by the crotonase-fold enzyme CarB/ThnE.^158^,^159^ CarB/ThnE have been shown to accept multiple substrate analogues and are amenable to engineering to produce multiple β-amino acids, many of which can be converted to β-lactams.^158^,^160^ In an analogous manner as occurring in the biosynthesis of clavulanic acid, but to give a bicycle, the carbapenem ring is formed *via* an ATP-dependent condensation catalysed by CarA/ThnM, forming (3*S*,5*S*)-carbapenam-3-carboxylic acid. The final steps in the biosynthesis of (5*R*)-carbapen-2-em-3-carboxylic acid are apparently catalysed by the 2OG-dependent oxygenase CarC. CarC catalyses the unusual epimerisation at the C-5 position, then desaturates the resultant (3*S*,5*R*)-carbapenam-3-carboxylic acid between C-2 and C-3 to give (5*R*)-carbapen-2-em-3-carboxylic acid (Fig. 12).^4^,^11^,^13^

Although recent studies have clarified key aspects of thienamycin biosynthesis, the overall thienamycin biosynthetic pathway is not fully elucidated. The biosynthetic gene cluster for thienamycin does not encode a close CarC homologue,^161^,^162^ and the mechanisms underlying carbapenam C-5 epimerisation and double bond formation in thienamycin biosynthesis are unclear. However, the pathways by which the C-2 and C-6 side chains are formed and functionalised have been elucidated, uncovering interesting chemistry involving 2OG oxygenases.^156^

Freeman *et al.* demonstrated that the C-2 cysteamine substituent of thienamycin is derived from coenzyme A *via* the action of hydrolytic enzymes ThnR, ThnH, and ThnT (Fig. 12).^157^ Whilst ThnR and ThnH sequentially degrade coenzyme A to pantetheine, ThnT degrades the pantetheinyl group to cysteamine, but only once it is covalently attached to the carbapenem core.^157^*N*-Acetylated thienamycin derivatives have been described; these are proposed to be formed through the action of the acetyltransferase ThnF.^157^ Further derivatives have been isolated which show desaturation of the C-2 cysteamine ethyl group and/or oxidation of the sulfur. Incubation of predicted substrates with the 2OG oxygenase ThnG showed that it catalyses sequential desaturation and sulfoxidation of the cysteamine side chain of deshydroxy *N*-acetylthienamycin (also referred to as PS-5; Fig. 12).^156^

The radical *S*-adenosylmethionine (SAM) enzyme ThnK is responsible for incorporation of the ethyl substituent at C-6 of thienamycin, in a mechanism involving two sequential radical-mediated methylation reactions.^163^ The 2OG oxygenase ThnQ has been shown to modify the C-6 ethyl group, by catalysing (8*R*)-hydroxylation (Fig. 12).^156^ Disruption of the ThnQ homolog involved in the biosynthesis of MM 4550 resulted in accumulation of an unhydroxylated product.^164^ Some carbapenems have different C-6 side chains, such as an (8*S*)-hydroxyl group or an (8*S*)-sulfate group (as seen in MM 4550).^4^ The epimerisation of the 8-hydroxyl group in MM 4550 is proposed to be catalysed by Cmm17, an enzyme homologous to the enoyl-CoA hydratases.^157^ The sulfotransferase CmmSu is proposed to catalyse sulfate formation, as supported by gene disruption studies.^164^

While these recent reports have greatly enhanced the understanding of the biosynthesis of the complex carbapenems, questions remain regarding some of the carbapenem biosynthetic steps. The mechanisms of desaturation and epimerisation of the five-membered ring in thienamycin biosynthesis are of particular interest. Further questions remain regarding the overall order in which modifications occur to the five-membered ring, the C-2, and the C-6 side chains. The enzymatic steps involved in the modifications seen in some other carbapenems (*e.g.*, the C-6 hydroxyisopropylidene group of the asparenomycins)^4^,^165^,^166^ also remain to be elucidated.

### 5.1. CarC – a carbapenem synthase

**Fig. 13:**
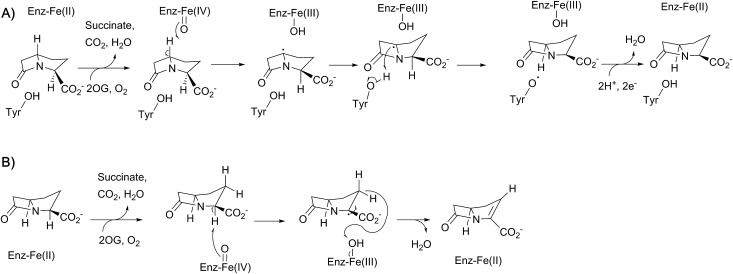
Proposed outline mechanisms for the two reactions involving CarC. (A) C5 inversion of stereochemistry; (B) oxidative C2–C3 desaturation.

In 1996, McGowan *et al.* described the gene cluster responsible for the biosynthesis of the simplest carbapenem (5*R*)-carbapen-2-em-3-carboxylic acid from *Erwinia carotovora* (now *Pectobacterium carotovorum*).^167^ In particular, homologies between CarA and CarC and the clavulanic acid biosynthetic enzymes β-lactam synthetase and CAS were noted.^167^ The role of CarC in carbapenem biosynthesis was supported the following year by mutational analysis.^168^ CarC is proposed to catalyse both epimerisation and desaturation of (3*S*,5*S*)-carbapenam-3-carboxylic acid, forming (5*R*)-carbapen-2-em-3-carboxylic acid [as well as (3*S*,5*R*)-carbapenam-3-carboxylic acid].^169^ Substrate studies demonstrated that (3*S*,5*R*)-carbapenam-3-carboxylic acid could also be converted to (5*R*)-carbapen-2-em-3-carboxylic acid by CarC, suggesting that the epimerisation activity of CarC precedes the desaturation activity (Fig. 13).^170^,^171^

**Fig. 14:**
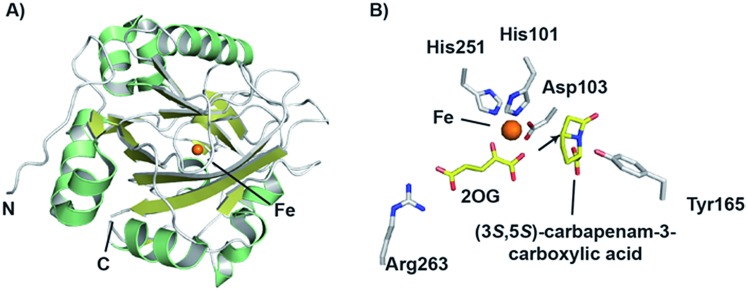
Views from structures of carbapenem synthase (CarC). (A) Overall view of a crystal structure of CarC (PDB 1NX4)^172^ showing the double-stranded β-helix fold. (B) View of the active site from a crystal structure of CarC with bound substrate (3*S*,5*S*)-carbapenam-3-carboxylic acid (PDB ; 4OJ8).^173^ Note that the C-5 hydrogen atom is likely abstracted by the ferryl iron species in the epimerisation step, with Tyr165 likely acting as the hydrogen atom donor on the other face.

Crystallographic studies of CarC have revealed the double-stranded β-helix core typical of the 2OG oxygenases.^172^ The active site Fe(ii) species was bound by a conserved 2-His-1-carboxylate triad as in IPNS/DAOCS, involving the side chains of His101, Asp103, and His251 (Fig. 14). 2OG was bound to the iron *via* the 2-keto and 1-carboxylate groups, while the C-5 carboxylate of 2OG forms an electrostatic interaction with the side chain of Arg263.^172^ Given the poor stability of (3*S*,5*S*)-carbapenam-3-carboxylic acid, a structure of CarC was first solved with bound l-*N*-acetylproline acting as a substrate analogue.^172^ While it was not possible to conclusively assign the orientation of l-*N*-acetylproline in this structure, modeling studies allowed the binding interactions of both (3*S*,5*S*)- and (3*S*,5*R*)-carbapenam-3-carboxylic acid with CarC to be studied.^172^ A later crystal structure of CarC was solved with bound (3*S*,5*S*)-carbapenam-3-carboxylic acid, in which two loops near the active site (which were disordered in previous structures) could be modeled.^173^ The orientation of (3*S*,5*S*)-carbapenam-3-carboxylic acid in this structure was such that the C-5 hydrogen atom was oriented towards the active site iron.^173^

Labeling studies, in which *Serratia marcescens* was treated with deuterium- and tritium-labeled l-prolines, indicated that an isotopic label is retained at the C-5 position of (3*S*,5*S*)-carbapenam-3-carboxylic acid, but lost at the C-5 position of the diastereomer (and initial CarC product) (3*S*,5*R*)-carbapenam-3-carboxylic acid.^174^ This led to the proposal that the epimerisation step involves the abstraction of the hydrogen atom at the C-5 position; computational studies suggested that formation of a C-5 radical was energetically favourable, most likely through hydrogen abstraction by the ferryl radical (followed by stereochemical inversion).^175^ Further density functional theory calculations and *in silico* docking studies were consistent with such a mechanism, and suggested that the phenol group of a tyrosine residue may act as a hydrogen atom donor on the other face of the C-5 radical, thereby completing the epimerisation step.^176^,^177^ Based on a crystal structure of CarC with substrate (3*S*,5*S*)-carbapenam-3-carboxylic acid (as well as mutational analysis), Tyr165 was proposed to play this role.^172^,^173^ The epimerisation step catalysed by CarC is redox neutral, and so it is possible that the desaturation step may employ the same ferryl species as that involved in the epimerisation step. However, rapid-kinetic and spectroscopic studies indicate that the epimerisation step is likely stoichiometric, occurring with oxidation of Fe(ii) to Fe(iii).^173^ Substitution of Tyr165 with a phenylalanine residue yielded an enzyme that catalyses catalytic substrate oxidation.^173^ Therefore, desaturation may require the generation of a new ferryl species, thus probably requiring a second catalytic cycle involving 2OG. The desaturation step, a more typical reaction catalysed by a 2OG oxygenase, may involve initial abstraction of a hydrogen atom at C-2.^173^

## 6. Potential roles of 2OG oxygenases in the biosynthesis of monocyclic β-lactams

Pioneering reports informed on the amino acid origins, role of peptide synthetases, and stereochemistry of ring formation in monocyclic β-lactam biosynthesis.^4^,^13^,^178^,^179^ However, until recently, relatively little has been described regarding the enzymology of the biosynthesis of monocyclic β-lactams. At least in the case of some monocyclic β-lactams, *i.e.* the monobactams and tabtoxin, sequencing studies have implicated 2OG oxygenases in their biosynthetic pathways, though likely not in actual β-lactam formation.^4^,^13^,^180^–^182^ Recent reports have informed on biosynthesis of the monocyclic β-lactams, highlighting chemically interesting mechanisms of cyclisation.^181^,^182^ Notably, whilst the β-lactam rings of the nocardicins^163^ and sulfazecin^182^ are both formed in reactions catalysed by non-ribosomal peptide synthetases (NRPS), the precise mechanisms of β-lactam formation differ for these two β-lactam antibiotics as described below.

The biosynthesis of nocardicin begins with NRPS proteins NocA and NocB.^181^,^183^ Based on the analysis of the five adenylation domains of these two proteins, a pentapeptide product/intermediate was predicted with a sequence of l-pHPG-l-Arg-d-pHPG-l-Ser-l-pHPG (pHPG, *p*-hydroxyphenylglycine).^183^ β-Lactam formation occurs while the nascent peptide chain is covalently attached to NocB (Fig. 3).^181^ β-Lactam formation was demonstrated to involve dehydration of the serine residue (as part of a NocB tetrapeptide peptide chain) bound to peptidyl carrier protein (PCP) domain PCP_4_, prior to the incorporation of the *C*-terminal pHPG residue. The α-amino group of this pHPG residue, while bound to PCP domain 5 as a thioester, can then undergo conjugate addition with the protein-linked dehydroalanine residue. The resultant secondary amino group of the PCP_5_-bound pHPG then attacks the thioester link to PCP_4_, forming the β-lactam and releasing the nascent peptide chain from PCP_4_. Finally, following the thioesterase-domain mediated hydrolysis, pro-nocardicin G is obtained; a further hydrolytic step and tailoring reactions lead to nocardicin A.^181^

While the β-lactam formation in nocardicin occurs on an NRPS PCP domain, the β-lactam ring of sulfazecin is proposed to occur *via* catalysis of an NRPS thioesterase domain.^182^ The NRPSs SulI and SulM, which contain three modules, generate an enzyme-bound tripeptide intermediate, consisting of a d-Glu residue (linked *via* its γ-carboxyl group), a d-Ala residue, and a l-2,3-diaminopropionate (Dap or β-aminoalanine) residue. The 3-amino group of Dap (in its SulM-bound tripeptide form) then undergoes an unusual *N*-sulfonation reaction, as catalysed by SulN, which uses 3′-phosphoadenosine 5′-phosphosulfate as a cosubstrate. The tripeptide is then transferred to a thioesterase domain, which catalyses nucleophilic attack of the sulfonated amino group onto the thioester carbonyl, thereby forming the β-lactam.^182^ This alternate strategy for β-lactam formation (*cf.* nocardicin) is possible as the β-lactam ring occurs at the *C*-terminus of the substrate, whereas that found in nocardicin occurs within the peptide sequence.

**Fig. 15:**
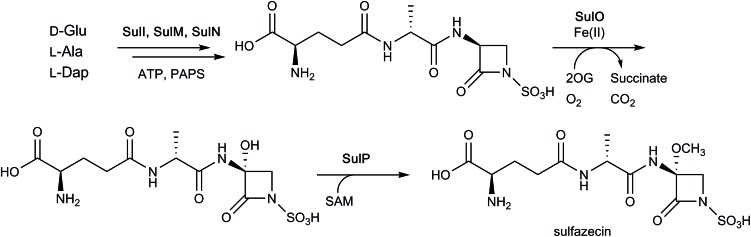
Final steps of sulfazecin biosynthesis. As shown in Fig. 3, the non-ribosomal peptide synthetases SulI and SulM are responsible for synthesising the sulfazecin peptide scaffold and β-lactam ring from d-glutamate, l-alanine, and l-2,3-diaminopropionate (l-Dap), while SulN acts as a sulfotransferase using 3′-phosphoadenosine 5′-phosphosulfate (PAPS) as a co-factor. The 2OG-dependent oxygenase SulO then catalyses C-3 hydroxylation, followed by *S*-adenosylmethionine (SAM)-mediated methylation as catalysed by SulP.^182^ Note that not all co-factors, co-substrates, and products are shown.

As seen with some cephalosporins (see above), the β-lactam rings of certain monocyclic β-lactams (such as sulfazecin and tabtoxin) manifest methoxylation at the C-3 position,^4^ a modification which can decrease susceptibility to some β-lactamases.^184^,^185^ The biosynthetic gene cluster associated with sulfazecin was noted to encode putative 2OG oxygenase, SulO, and methyltransferase, SulP.^184^,^185^ As indicated above, the NRPSs SulI and SulM and sulfotransferase SulN catalyse the formation of desmethoxysulfazecin. Addition of SulO and SulP to this *in vitro* reconstitution of the biosynthetic pathway (with added 2OG, ammonium ferrous sulfate, and ascorbate) yielded sulfazecin, confirming the role of SulO in monobactam C-3 hydroxylation (Fig. 15).^182^ The tabtoxin gene cluster encodes for putative 2OG oxygenase TblC, which likely plays an analogous role in tabtoxin biosynthesis.^186^

## 7. Conclusions

In the period following Arnstein's breakthrough proposal^26^ for the peptide origin of the penicillins and Abraham's direct evidence^27^ for the role of l,l,d-ACV (and by implication an oxidative mechanism for penicillin formation) very considerable advances have been made in our understanding of β-lactam biosynthesis. IPNS was isolated and shown to operate *via* a chemically remarkable mechanism.^22^ ATP dependent synthetases that catalyse β-lactam formation from β-amino acids have been identified and peptide synthetase templated β-lactam formation *via* thioester reactions^180^,^181^ has been shown to operate for at least two classes of monocyclic β-lactams. Aside from the central role of IPNS, 2OG oxygenases have been shown to play roles in the biosynthesis of bicyclic β-lactams (DAOCS) and in modifying both monocyclic and bicyclic β-lactams, sometimes by chemically interesting processes (especially the DAOCS, CAS, and CarC reactions).^4^ The biosynthetic work, including on the 2OG oxygenase superfamily members, has been justified in part on the basis that it may help enable improved routes to already used antibiotics (*e.g.* by enabling fermentation of carbapenems) and make synthetically challenging structures of interest for testing as antibiotics more accessible. Despite ongoing efforts to engineer cephalosporin biosynthesis, these ‘translational’ outcomes have yet to be realised. This is likely in part due to commercial and regulatory considerations associated with the introduction of new manufacturing procedures for (new) antibiotics, coupled with the success of classical empirical strain optimisation and lack of knowledge of ‘indirect’ limiting molecular factors for the highly efficient large scale fermentation and isolation of relatively labile small molecules. Recent work on metabolic engineering, *e.g.* TCA cycle glyoxylate metabolism (as well as deleting β-lactamases) holds promise for increasing β-lactam antibiotic production yields.^119^,^187^,^188^

Combining these approaches with biosynthetic pathway engineering may be productive. The results to date on β-lactam biosynthesis do, however, mean that focused efforts to engineer β-lactam biosynthesis, *e.g.* by ‘rewiring’ the penicillin/cephalosporin pathway to produce new generation cephalosporins *via* modification of ACVS/IPNS/DAOCS, could at least proceed in the light of detailed knowledge of how the relevant enzymes work. Future biocatalytic research could also explore the use of modified enzymes in combination with non-enzymatic synthetic chemistry to access modified β-lactams of interest in the field of antibacterials and beyond. Engineering involving coupling the modular nature of peptide synthetase mediated β-lactam biosynthesis with the tailoring capabilities of 2OG oxygenases is one potential avenue.

It is, however, important to note that although the basic work on the enzymes of β-lactam biosynthesis has not (yet) delivered breakthroughs in the antibacterial field, the chemical interest driven work on enzyme mechanisms and structures has been enabling in other fields. The structural studies on IPNS and the β-lactam modifying 2OG oxygenases^28^–^30^,^145^ led to the identification of apparently homologous enzymes in most aerobic organisms. Some of these are used in biocatalysis, others are important in plant signalling (‘ethylene-forming-enzymes’) and as agrochemical targets (gibberellin biosynthesis oxygenases), whilst many others play crucial roles in animal biology, notably in post translational protein modifications and in the regulation of nucleic acid and protein biosynthesis.^22^ Inhibitors for human 2OG dependent prolyl-hydroxylases, which play a critical role in the cellular response to hypoxia, are presently in late stage clinical trials for the treatment of anaemia.^189^ Hence, at least, the work on the oxygenases of β-lactam biosynthesis is a nice example of how curiosity driven research on chemically interesting natural products can help open new fields of medicinal relevance.

## 8. Conflicts of interest

The authors declare no conflicts of interest.
